# Identification, cloning and characterization of the tomato TCP transcription factor family

**DOI:** 10.1186/1471-2229-14-157

**Published:** 2014-06-06

**Authors:** Violeta Parapunova, Marco Busscher, Jacqueline Busscher-Lange, Michiel Lammers, Rumyana Karlova, Arnaud G Bovy, Gerco C Angenent, Ruud A de Maagd

**Affiliations:** 1 Laboratory of Molecular Biology, Department of Plant Sciences, Wageningen-UR, PO Box 386, 6700 AJ Wageningen, the Netherlands; 2 Plant Research International, P.O. Box 619, 6700 AP Wageningen, the Netherlands; 3 Molecular Plant Physiology, Utrecht University, Padualaan 8, 3584 CH Utrecht, the Netherlands

**Keywords:** Transcription factors, Tomato, Yeast one-hybrid, Yeast two-hybrid

## Abstract

**Background:**

TCP proteins are plant-specific transcription factors, which are known to have a wide range of functions in different plant species such as in leaf development, flower symmetry, shoot branching, and senescence. Only a small number of *TCP* genes has been characterised from tomato (*Solanum lycopersicum*). Here we report several functional features of the members of the entire family present in the tomato genome.

**Results:**

We have identified 30 *Solanum lycopersicum SlTCP* genes, most of which have not been described before. Phylogenetic analysis clearly distinguishes two homology classes of the SlTCP transcription factor family - class I and class II. Class II differentiates in two subclasses, the CIN-TCP subclass and the CYC/TB1 subclass, involved in leaf development and axillary shoots formation, respectively. The expression patterns of all members were determined by quantitative PCR. Several *SlTCP* genes, like *SlTCP12*, *SlTCP15* and *SlTCP18* are preferentially expressed in the tomato fruit, suggesting a role during fruit development or ripening. These genes are regulated by RIN (RIPENING INHIBITOR), CNR (COLORLESS NON-RIPENING) and SlAP2a (APETALA2a) proteins, which are transcription factors with key roles in ripening. With a yeast one-hybrid assay we demonstrated that RIN binds the promoter fragments of *SlTCP12*, *SlTCP15* and *SlTCP18*, and that CNR binds the *SlTCP18* promoter. This data strongly suggests that these class I SlTCP proteins are involved in ripening. Furthermore, we demonstrate that SlTCPs bind the promoter fragments of members of their own family, indicating that they regulate each other. Additional yeast one-hybrid studies performed with *Arabidopsis* transcription factors revealed binding of the promoter fragments by proteins involved in the ethylene signal transduction pathway, contributing to the idea that these *SlTCP* genes are involved in the ripening process. Yeast two-hybrid data shows that SlTCP proteins can form homo and heterodimers, suggesting that they act together in order to form functional protein complexes and together regulate developmental processes in tomato.

**Conclusions:**

The comprehensive analysis we performed, like phylogenetic analysis, expression studies, identification of the upstream regulators and the dimerization specificity of the tomato TCP transcription factor family provides the basis for functional studies to reveal the role of this family in tomato development.

## Background

TCP proteins, named after the three first characterized family members TEOSINTE BRANCHED (TB) 1 from maize, CYCLOIDEA (CYC) from *Antirrhinum majus*, and PROLIFERATING CELL FACTORS (PCFs) from rice are plant-specific transcription factors characterized by the TCP domain, a motif encompassing a non-canonical basic-helix-loop-helix (bHLH) structure [1]. While initially these transcription factors were implicated in the regulation of growth and development, it has become apparent that they are involved in many processes including senescence, circadian rhythm and hormone signaling (reviewed in: [2,3]). Based on the homology of the TCP domains, TCP proteins can be divided into two major classes, class I and class II. The first is represented by the rice PCF proteins and many of its members have no known function so far. The Arabidopsis gene *AtTCP14* has been shown to regulate embryonic growth potential in Arabidopsis seeds [4] and together with *AtTCP15*, it regulates internode length [5]. AtTCP20 appears to function in diverse developmental processes, such as growth processes [6], jasmonic acid (JA) biosynthesis and leaf senescence [7]. *AtTCP16* is predominantly expressed in developing microspores, and its down-regulation in transgenic plants resulted in 50% abnormal pollen [8].

Class II, represented by *CYC* and *TB1*, contains most genes with known functions. The *CYC* gene, together with the related *DICHOTOMA* (*DICH*) in *Antirrhinum* is required for dorsoventral asymmetry of the flower [9]. The Arabidopsis *CYC/DICH* homolog *AtTCP1* regulates the expression of the brassinosteroid synthetic gene *DWARF4* and is thus also linked to growth [10]. The *TB1* gene affects the fate of maize axillary meristems. It prevents the outgrowth of buds at the lower nodes and it promotes the formation of female inflorescences at the higher nodes [11]. In Arabidopsis, two homologs of *TB1*, *BRANCHED1* (*BRC1, AtTCP18*) and *BRANCHED2 (BRC2, AtTCP12)* are expressed in axillary buds, and mutants with reduced activity of either gene show increased branching [12]. The tomato orthologs *SlBRC1a* and *SlBRC1b* have similar functions in tomato axillary bud initiation and outgrowth [13].

Other examples of *TCP* genes affecting plant architecture are *CINCINATTA* in *Antirrhinum*[14] and its homolog *LANCEOLATE* (*LA, SlTCP2*) in tomato [15]. The dominant *Lanceolate* mutation in tomato produces small simple leaves instead of the normally large and compound ones. The corresponding *TCP* gene allele *la* contains point mutations in a *miR319*-binding site, leading to reduced sensitivity to miRNA regulation. One other full-length mRNA and two mRNA fragments with putative *miR319*-binding sites, designated *SlTCP3*, *SlTCP10* and *SlTCP24* after their closest homologs in Arabidopsis, respectively, were also identified [15]. The Arabidopsis homologs, as well as *AtTCP2* and *AtTCP4* (the closest homologs of *LANCEOLATE* and *CIN*) have been earlier identified as targets of *miRNA319* through activation-tagging mutants of the *miRNA319*-encoding *JAW* locus [16] and are therefore, called *JAW* clade *TCP* genes. In the activation-tagged *jawD* mutant, which exhibits a reduced expression of the *JAW* clade *TCP* genes, the differential regulation of cell division during leaf development is disturbed, causing negative leaf curvature and crinkly leaves [17,18]. *MiRNA319*-targetting *AtTCP4* is required for petal growth and development [19]. The same clade of *miRNA319*-regulated genes in Arabidopsis has been shown to control JA synthesis and leaf senescence [20]. Studies in Arabidopsis suggest that *CCA1 HIKING EXPEDITION (CHE) (AtTCP21)* is involved in circadian clock regulation by repressing the *CIRCADIAN CLOCK ASSOCIATED 1 (CCA1)* gene [21].

A group of homologous TCPs of Arabidopsis functions redundantly in the control of shoot lateral organ morphology through the negative regulation of boundary-specific genes such as *CUP-SHAPED COTELYDON 1*[22]. The only known example of a *TCP* gene affecting fruit development is the phenotype of a dominant-negative variant of *AtTCP3*, where the siliques are shorter and their surface wrinkled [22].

As in other transcription factors, the basic region of the TCP domain is likely to be involved in DNA binding, but deletion studies have shown that both N- as well as C-terminal regions of the conserved bHLH-domain are required for target site recognition by the rice PCF1 [23]. Modeling of Arabidopsis TCP4 dimers bound to target DNA suggest that the first part of the basic region of the TCP domain forms a small α-helix involved in DNA base interactions and the C-terminal part of this region may form an α-helix contiguous with Helix 1. Homology modeling based on the animal bHLH protein myoD suggests that the Helix-loop-helix region is responsible for dimerization [24]. It was shown that TCP proteins tend to form homodimers or heterodimers with other TCP proteins of the same class [25].

The consensus binding site sequences for the two classes are distinct, but overlapping (GGNCCCAC for class I and GTGGNCCC for class II) and the core (GGNCCC) shared by these sequences has a strict role in the binding of both classes [25]. TCP-binding elements are found in the promoters of various cell cycle related genes and of genes encoding ribosomal proteins [26]. AtTCP20 has been found to physically bind to synthetic versions of these elements, as well as to *cis*-elements in the promoter of the mitotic cyclin *CYCB1;1* gene. It was proposed that organ growth rates and possibly shape are regulated by the balance between positively and negatively acting TCP proteins competing for binding to the same promoters [27]. Later, it was found that class I TCP proteins act antagonistically to the class II *JAW*-TCPs via the JA signaling pathway, as TCP20 inhibits *LOX2 (LIPOXYGENASE 2)* and TCP4 induces *LOX2* expression [7].

In this manuscript we describe the identification and characterization of 30 different TCP-encoding genes from tomato (*Solanum lycopersicum*). Using quantitative RT-PCR we have determined their expression in different tissues and during fruit development, revealing differential expression patterns of members during fruit development and ripening. The latter was shown to be dependent on several major ripening regulatory transcription factors like LeMADS-RIN (RIPENING-INHIBITOR) [28], a MADS box protein, COLORLESS NON-RIPENING (CNR), a SQUAMOSA promoter binding protein (SBP) [29], and APETALA2a (SlAP2a), an APETALA2/ETHYLENE RESPONSE FACTOR (AP2/ERF) [30]*.* These ripening-associated transcription factors regulate ripening through the biosynthesis of ethylene and/or its signalling.

This is the first study revealing the correlation of TCP transcription factors in fleshy fruit development and ripening. Moreover, we further investigated their regulation by identification of transcription factors interacting with promoter sequences of these genes in a yeast one-hybrid assay. Furthermore, in a yeast 2-hybrid assay we have determined the capacity of the tomato TCP proteins to form homo- and heterodimeric interactions. Comparison of the characteristics of the tomato family members with those from other plant species may reveal common and diverged features and may give clues about the function of the tomato *TCP* genes.

## Results and discussion

### Identification and cloning of tomato TCP genes

By mining the tomato Unigene and BAC sequence databases from the Sol Genomics Network with homology searches and subsequent sequence extension by RACE, we initially identified and cloned 24 different tomato genes encoding putative TCP transcription factors (Table 1; *SlTCP1-24*). Many of the sequences we have identified had only 1 or few representative EST in the databases. Four genes had no representative EST and were identified directly from the genomic sequence available at the time. Publication of the tomato genome sequence allowed the identification of another 6 *SlTCP* genes (Table 1). Of the 30 identified unique genes, 2 full-length mRNA sequences, for *Lanceolate* and *SlTCP3*[15], and three partial sequences, previously named *SlTCP1*, *SlTCP2*, and *SlTCP3*[31], here renamed *SlTCP22*, *SlTCP7*, and *SlTCP8*, respectively, were already present in Genbank. *SlTCP7* and *SlTCP9*[31] were subsequently also named *BRC1B* and *BRC1A*, respectively [13].

**Table 1:** Tomato TCP genes

**Name**	**iTAG2.3**	**Previous/alternate name**	**Accession no.**
***SlTCP1***	Solyc02g077250.2		GQ496320
***SlTCP2***	Solyc07g062680.1	*Cycloidea/Lanceolate*	AF175965
***SlTCP3***	Solyc12g014140.1	*SlTCP3* (fragment)	GQ496321
***SlTCP4***	Solyc03g115010.1		GQ496322
***SlTCP5***	Solyc02g089020.1		GQ496323
***SlTCP6***	Solyc06g069460.1		GQ496324
***SlTCP7***	Solyc02g089830.1	*SlTCP2* (fragment)/*BRC1B*	GQ496325
***SlTCP8***	Solyc06g069240.1	*SlTCP3* (fragment)	GQ496326
***SlTCP9***	Solyc03g119770.2	*BRC1A*	GQ496327
***SlTCP10***	Solyc07g053410.2		GQ496328
***SlTCP11***	Solyc01g103780.2		GQ496329
***SlTCP12***	Solyc11g020670.1		GQ496330
***SlTCP13***	Solyc06g065190.1		GQ496331
***SlTCP14***	Solyc04g009180.1		GQ496332
***SlTCP15***	Solyc01g008230.2		GQ496333
***SlTCP16***	Solyc03g116320.2		GQ496334
***SlTCP17***	Solyc06g070900.2		GQ496335
***SlTCP18***	Solyc02g068200.1		GQ496336
***SlTCP19***	Solyc09g008030.1		GQ496337
***SlTCP20***	Solyc08g080150.1		GQ496338
***SlTCP21***	Solyc03g006800.1		GQ496339
***SlTCP22***	Solyc04g006980.1	*SlTCP1* (fragment)	GQ496342
***SlTCP23***	Solyc05g007420.1		GQ496340
***SlTCP24***	Solyc08g048390.1		GQ496341
***SlTCP25***	Solyc05g009900.1		
***SlTCP26***	Solyc03g045030.1		
***SlTCP27***	Solyc02g094290.1		
***SlTCP28***	Solyc02g065800.1		
***SlTCP29***	Solyc08g048370.2		
***SlTCP30***	Solyc10g008780.1		

### Genomic organization and phylogenetic analysis

The chromosome location of the 30 genes is depicted in Additional file 1: Figure S1. We found in the published Heinz 1706 genome (v2.40) four additional full length copies of *SlTCP1(named TCP1a-d)* and one partial copy each of *SlTCP19 (a)* and *SlTCP28 (a)*, respectively, in different genomic locations (listed in Additional file 2: Table S1 and shown in Additional file 1: Figure S1). The additional copies of *SlTCP1* have in their close vicinity open reading frames with homology to transposon sequences, suggesting that they are the result of mobilization by transposable element activity. Since our experimentally determined mRNA sequences as well as publicly available EST sequences map uniquely to the respective genes and not to these additional copies, we conclude that these copies are not expressed under conditions used by ourselves or by others. This is further supported by the observation of genomic synteny in the Plant Genome Duplication Database (PGDD) [32], where *SlTCP1* and its genomic environment is contained in a block showing extensive synteny with 22 different genomic sequence blocks in 11 plant species, with the four copies (*SlTCP1a-d*) showing no synteny with other genomes at all (not shown). Similarly, there is an almost perfect copy (3 mismatches in 529 nucleotides) of *SlTCP28*, which is also associated with transposon-like sequences.

**Figure 1:**
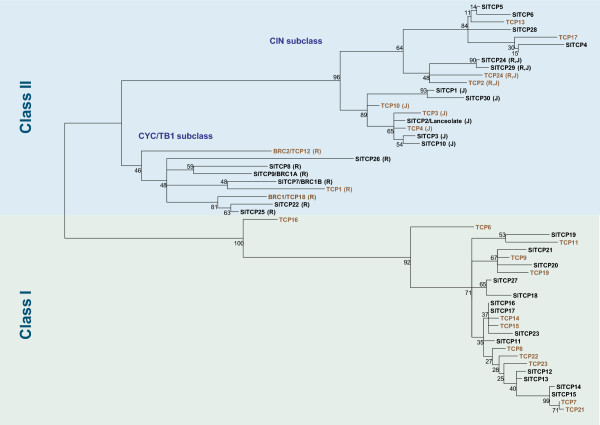
**Protein phylogenetic tree of the extended TCP domains of tomato and Arabidopsis TCP proteins.** Arabidopsis proteins are indicated in brown. Proteins marked with “J” have mRNA’s with (putative) target sites for jaw (miR319a) and those marked with “R” have an R-domain.

Phylogenetic analysis of the extended TCP domains of the predicted proteins following alignment together with the 24 known Arabidopsis TCP proteins is shown in Figure 1. The phylogenetic comparison with the Arabidopsis TCP proteins showed that conservation between proteins of the two species is usually low. Higher similarity of proteins within the same species indicates that gene duplications have occurred after the split between the two lineages. It also suggests that the higher number of genes in tomato, compared to *Arabidopsis*, is the result of more gene duplication events in tomato or of higher frequency of retaining copies after duplication. Only the Arabidopsis AtTCP16 stands out as not having a close homolog in tomato.

**Figure 2:**
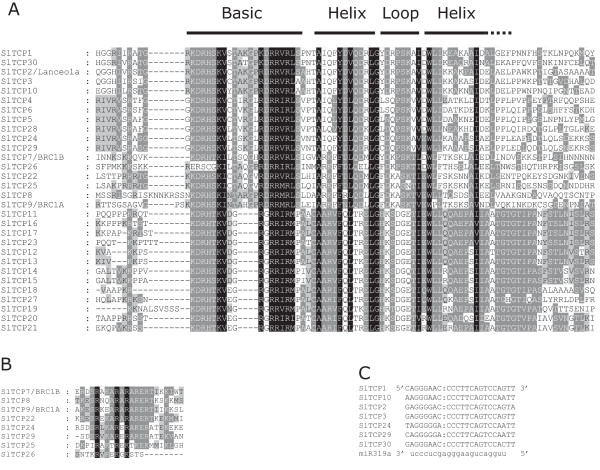
**TCP protein sequence alignments. A**. Alignment of the TCP domain and adjoining sequence for the predicted tomato TCP proteins. Overall conserved amino acids are shaded in black. Amino acids over 80% conserved in Class II or Class I are shaded in light gray and dark gray, respectively. The Basic, Helix I, Loop, and Helix II regions are indicated. **B**. Alignment of the R-domain of Class II subfamily members. Sequences were aligned with ClustalW and visualized with Genedoc. **C**. Alignment of putative target areas for miR319a (aligned in reverse).

The phylogenetic analysis showed that sequence conservation outside the approximately 60 amino acid TCP domain was low and sequence length on both sides of the TCP domain varied greatly, resulting in proteins ranging from 113 (TCP27) to 409 amino acids. The smallest predicted protein, SlTCP27, is probably truncated by a frame shift mutation since sequence homology with Arabidopsis TCP20 extends well beyond the stop codon. The low overall conservation resulted in relatively low bootstrap values, indicating poor reliability of some of the branches. Analysis of the phylogenetic tree as well as of the alignment of the TCP domains (Figure 2A) showed that tomato TCP proteins can be divided into two subfamilies, as for all species so far. The CYC/TB1 or class II subfamily has, as reported earlier, an extended basic region, while class I subfamily members have extended homology C-terminal from the TCP domain, and both subfamilies have internally conserved, but distinct loop region sequences [1]. The phylogenetic tree also supported the Arabidopsis and rice earlier described division of class II proteins in two further subfamilies [33]. According to this division, class IIa or CYC/TB1 contains the tomato genes *BRC1B* (*SlTCP7*), *SlTCP8*, *BRC1A* (*SlTCP9*), *SlTCP22*, *SlTCP25*, and *SlTCP26*. From Figure 2A it is evident that SlTCP26 lacks the conserved N-terminal part of the basic region, which suggests that this protein may not be able to bind DNA.

Class IIb or CIN-TCPs, so named after their homology with *Antirrhinum* CINCINATTA, is a group of 8 TCP proteins in Arabidopsis involved in leaf growth regulation (AtTCP2, -3, -4, -5, -10, -13, -17, and -24) [15,16]. Tomato has 11 proteins in this homology group, among which the earlier identified LANCEOLATE, SlTCP1 to SlTCP6, SlTCP10, -24, -28, -29 and -30 (Figure 1). The tree topology, particularly for class II, was further supported by analysis of synteny. Inspection of synteny in the Plant Genome Duplication Database (PGDD) found synteny between genomic regions for all tomato and Arabidopsis genes in the CYC/TB1-subclass, as well as for the genes from the subclade containing *Lanceolate* and the subclade containing tomato *TCP4*, -*5*, -*6*, and -*28*. This suggests that members from these subclades originated from ancient whole genome or segmental duplications in a common ancestor of Arabidopsis and tomato. Tomato *TCP24* and -*29* do not show any synteny in the PGDD, whereas their closest Arabidopsis homologs do have extensive synteny with various species. Since these genes are arranged as a tandem inverted repeat on chromosome 8, they may be the result of a lineage-specific rearrangement that is absent in Arabidopsis.

As reported earlier for Arabidopsis TCP1, TCP2, TCP12, TCP18, and TCP24, a subset of the closest tomato homologs of these Arabidopsis TCP proteins, BRC1B (SlTCP7), SlTCP8, BRC1A (SlTCP9), SlTCP22, SlTCP24, and SlTCP29 contain the so-called R-domain C-terminal of the TCP domain [33]. R domain-like sequences are also present in SlTCP25 and SlTCP26, but there they are less conserved (Figure 2B).

In Arabidopsis, 5 of the class IIb members are post-transcriptionally regulated by *miRNA319* (*AtTCP2, 3, 4, 10*, and *24*) [15,16,18]. The closest tomato homologs of these Arabidopsis genes are the three new genes, *SlTCP1*, *SlTCP29*, and *SlTCP30*, and the earlier identified *SlTCP10*, *LANCEOLATE (SlTCP2)*, *SlTCP3* and *SlTCP24*, respectively [15]), all having a putative binding site for *miR319a*. This suggests that regulation of leaf development by a redundant set of miRNA-regulated homologous *TCP* genes occurs in tomato. *SlTCP2, -3, -10, -24, and -30* have considerable expression, although not exclusively, in tomato leaves. Figure 2C shows the alignment of the target sites of these genes with the *miR319a* sequence. In a previously published degradome study, the transcripts of all putative *miR319* targets identified here, with exception of *SlTCP30*, were shown to be actually cleaved in tomato fruits [34].

### Expression analysis of the tomato TCP genes

**Figure 3:**
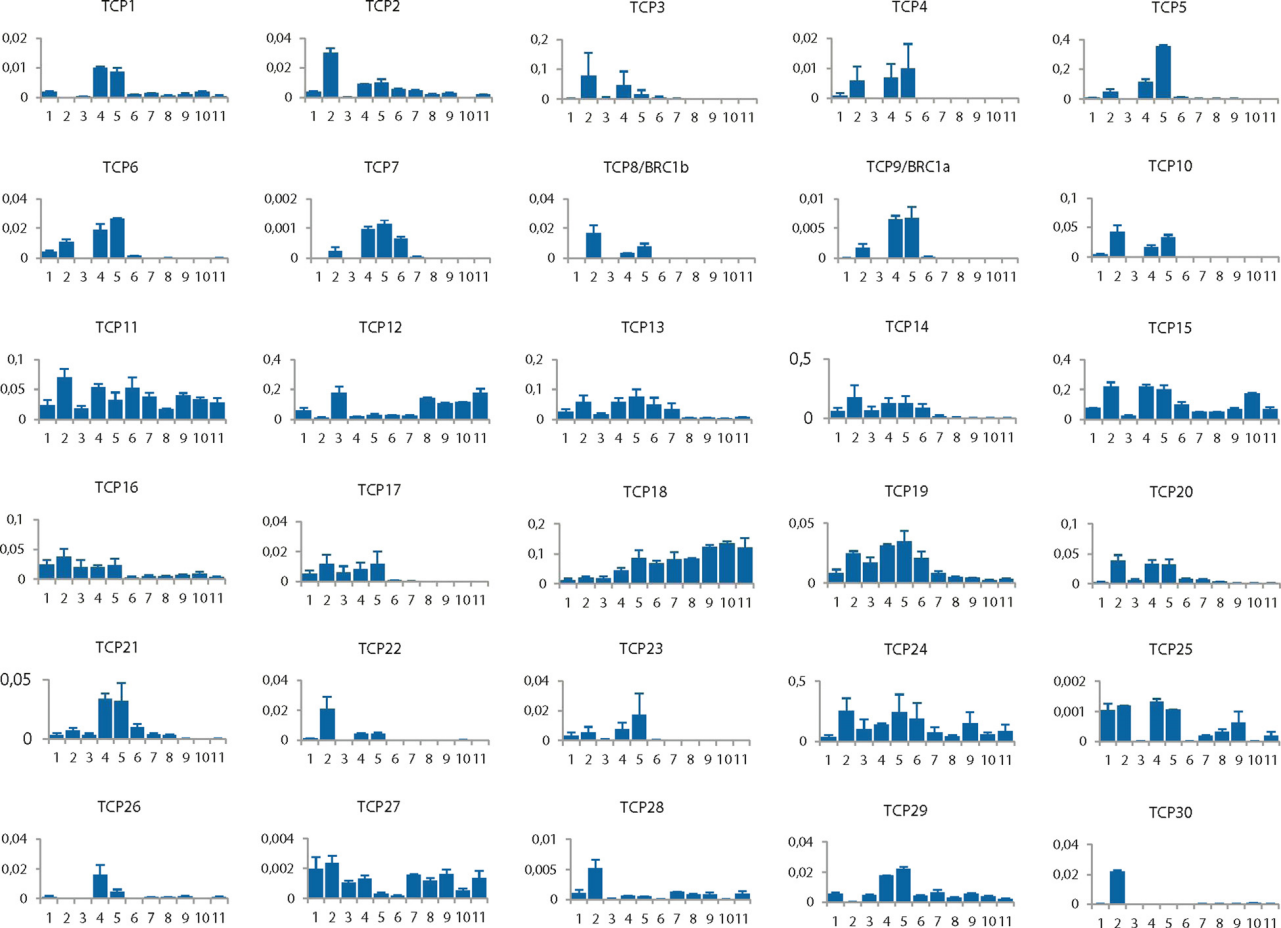
**Expression of tomato TCP genes.** Relative transcript levels, determined by quantitative real time RT-PCR, relative to the expression of the β-actin gene, expressed as 2 ^–δCt^. Tissue samples are coded by number. 1: seedlings; 2: leaves; 3: roots; 4: flowers at anthesis; 5: flowers 2 DPA; 6: fruits 5 mm diameter. 7: fruits 18 mm diameter. 8: mature green fruit; 9: breaker stage fruit; 10: turning stage fruit; 11: red ripe fruit.

In order to predict possible functions as well as to identify probable functional redundancy through overlapping expression patterns for the tomato *TCP* genes, we determined expression levels of all 30 genes by quantitative RT-PCR. We used mRNA isolated from: tomato seedlings, leaves, roots, flowers at anthesis, flowers at 2 days post anthesis (DPA), immature green fruit at two sizes (5 mm diameter and 18 mm diameter, respectively), mature green fruit, breaker stage fruit, turning stage fruit, and red ripe fruit. All expression levels (in this order) as related to the expression of the β-actin gene are shown in numerical order in Figure 3. From Figure 3 it is apparent that the expression levels in different organs vary widely between the tomato *TCP* genes, as well as between different organs for individual *TCP* genes. The representatives of class II TCPs have high expression mostly in flowers at anthesis, 2 DPA and leaves. In addition to the already mentioned organs, *SlTCP25* shows relatively high expression in seedlings and lower in developing fruits.

We have identified 13 proteins as member of the class I group. Many class I genes seem more widely and less specifically expressed, such as in leaves, flowers, and early stages of developing fruits (Figure 3). *SlTCP11 and TCP27* appear to lack organ specificity, while *SlTCP12* and *SlTCP18* are the only genes with high expression during ripening, with *SlTCP12* particularly rising at and beyond the mature green stage. Most class II genes are not expressed in fruits beyond the flower 2 DPA stage. *SlTCP15* is relatively highly expressed up to the 5 mm fruit stage, after which the expression drops and comes back during ripening, with highest expression in the turning stage. The expression in tomato fruit is of particular interest since this is the first example of extensive characterization of TCP gene expression in a fleshy fruit species. *SlTCP27* is regulated during ripening; however its expression is low in all tissues. *SlTCP12* and *SlTCP18* are particularly interesting because of their expression during fruit ripening. The expression pattern of *SlTCP12* is strikingly complementary to that of its closest homolog *SlTCP13*, possibly pointing to an example of neofunctionalisation after gene duplication through divergence of expression patterns. Opposite to the upward regulation of *SlTCP12* during ripening, SlTCP13, -14, -15 and -19 show high expression up to the 5 mm fruit stage, followed by a sharp drop and *SlTCP15* being expressed again during ripening. The regulation of *SlTCP12, -15* and -*18* during the onset of ripening may have functional significance that may not apply in non-fleshy Arabidopsis siliques.

### Interactions between tomato TCP proteins

**Figure 4:**
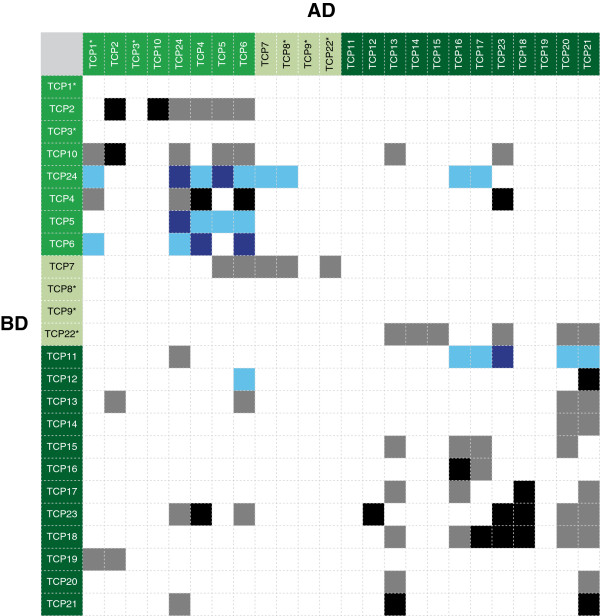
**Interaction of tomato TCP proteins in a yeast two-hybrid assay.** BD-fusions are listed in the left column. TCP protein names are ordered according to their subclass (highlighted green, light-green and dark-green, respectively). BD-fusions exhibiting autoactivation are marked with an asterisk. Interactions only scored on –LTA medium are shown in blue. Interactions found in both directions (BD/AD and AD/BD) are depicted as a black or dark-blue, one-way interactions in gray and light blue, respectively. Scoring tables of the yeast 2-hybrid experiments can be found in Additional file 3: Table S2.

TCP proteins tend to form homodimers or heterodimers with other TCP proteins, and dimerization may be required for their DNA-binding activity and hence for their biological activity. We have determined dimer formation between 24 cloned tomato SlTCP proteins in a yeast 2-hybrid assay. Open reading frames were cloned as translational fusions with the yeast GAL4 transcription factor binding- (BD-) or activation- (AD-) domain and all combinations were tested in a matrix set-up. Results are represented schematically in Figure 4, where the proteins are arranged according to their phylogenetic relatedness, and interaction scoring tables can be found in Additional file 3: Table S2. Of the 24 BD-fusion proteins tested, 5 had autoactivation activity in yeast (highlighted with asterisk in Figure 4) on both selection media, while four showed autoactivation only on -LTH medium. With the exception of SlTCP12, these were found to be all class II TCP transcription factors, an overrepresentation that was also observed for Arabidopsis TCP transcription factors [7]. Thus, interactions could not be scored for the corresponding BD clones (empty rows in Figure 4) or could be scored only on –LTA medium. Altogether we observed 92 interactions, with a few exceptions on both selective media. Of these, in 34 (17 pairs) the partners interacted in both BD/AD-orientations, including 6 homodimer formations. The latter number may be an underestimation because homodimer formation could not be tested in the autoactivating family members. The summary of the interaction results shows that tomato TCP proteins form both homo- and heterodimers, in the latter case preferentially with proteins of the same class (77 interactions) as was previously noted for Arabidopsis [35], although a few (15) inter-class interactions were detected. SlTCP12, which shows autoactivating activity on one medium, has only SlTCP6 and SlTCP21 overlapping and five different interactions compared to its nearest homolog SlTCP13, which is not autoactivating. Together with the different expression patterns, this points to functional divergence after the gene duplication. Again similar to the Arabidopsis TCP proteins, more interactions were found for class I proteins than were found for Class II proteins (42 versus 33), although also here the number of interactions for Class II proteins may be underestimated because of the autoactivating members. The interactions obtained by a comprehensive yeast two-hybrid screen of the tomato TCP transcription factors, has not yet been reported to such extent for TCP members from other species than Arabidopsis. These yeast 2-hybrid interactions of the tomato TCP genes confirmed earlier observations for rice [23,25], showing that TCP proteins form homodimers, and heterodimers particularly with proteins within the same class. The combination of expression analysis and dimerization properties may in the future help to identify TCP protein pairs that function together and to explain observed functional redundancies in case of overlapping interaction maps.

### The expression of the tomato *TCP12*, *TCP15* and *TCP18* genes is affected by mutations in major ripening regulatory genes

**Figure 5:**
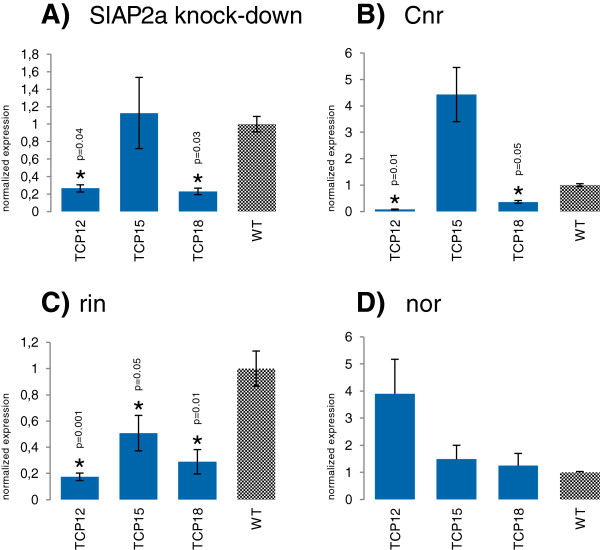
**Normalized expression of *SlTCP12*, *SlTCP15 *and *SlTCP18*in tomato fruit at Breaker + 7 stage.** Expression values are normalized for each gene related to wild type, expressed as 2 ^–δδCt^. **A**. In the ripening defective transgenic plants with RNAi-suppressed *SlAP2a. T*-test p-values: 0.04 (*SlTCP12*), 0.81 (*SlTCP15*), and 0.03 (*SlTCP18*). **B**. In the ripening defective mutant *Cnr*, p-values: 0.01 (*SlTCP12*), 0.19 (*SlTCP15*), and 0.05 (*SlTCP18*). **C**. In the ripening defective mutant *rin*, p-values: 0.001 (*SlTCP12*), 0.05 (*SlTCP15*), and 0.01 (*SlTCP18*). **D**. In the ripening defective mutant *nor*, p-values: 0.3 (*SlTCP12*), 0.5 (*SlTCP15*), and 0.7 (*SlTCP18*). Asterisks indicate significant differences (p ≤ 0.05).

We demonstrated that *SlTCP12*, and -*18* show differential expression during fruit ripening, with *SlTCP12* highly expressed in the ripening stages and *SlTCP18* being increasingly expressed from early stages gradually to red ripe fruits. *SlTCP15* expression is high in flowers at anthesis, 2 DPA and 5 mm fruit, which are stages associated with a high mitotic index rate [36] and then regulated again during ripening, peaking in the breaker stage. The expression patterns of these TCPs suggested that they might be positively or negatively regulated by ripening, and thus directly or indirectly by some of the known major regulators of this process. We determined the expression of these *TCP* genes by qRT-PCR analysis performed in fruits of the Br + 7 (7 days after the breaker) stage of the tomato ripening-defective mutants *Cnr (Colorless non-ripening)*[37]*, rin (ripening-Inhibitor)*[38]*, nor*[39] and in transgenic *SlAP2a* knock-down plants [40]. As expected, the transcript levels of Sl*TCP12, SlTCP15* and *SlTCP18* are regulated in several of the ripening mutants (Figure 5A, 5B, 5C). *SlTCP12*, associated with ripening, is significantly and positively regulated by *SlAP2a, CNR* and *RIN* (Figure 5A, 5B, 5C), while the expression of *SlTCP15*, associated with early fruit development, is not statistically significantly regulated by *SlAP2a* and *CNR* (Figure 5A, 5B). RIN positively and significantly regulates *SlTCP15* (Figure 5C). *SlTCP18*, which has expression in all stages of fruit development, with a high increase during ripening, is significantly down regulated in *SlAP2a* RNAi, *Cnr* and *rin* (Figure 5A, 5B, 5C). The three TCPs are not significantly regulated in the *nor* mutant (Figure 5D). The positive regulation of *SlTCP12* and *SlTCP18* by *CNR*, *SlAP2a* and *RIN*, indicates that they might be positively associated with ripening. Additionally, *RIN* positively regulates *SlTCP15*, associating it also with the ripening process.

### Ripening-related tomato transcription factor proteins bind *SlTCP12,-15* and -*18* promoter elements

The altered expression of *SlTCP12, -15* and -*18* in the ripening mutants and *SlAP2a* transgenic knockdown plants indicates that they are regulated by key ripening regulatory genes, but does not show if this regulation is direct. In order to find out if these transcription factors could be directly interacting with the promoters of *SlTCP12, SlTCP15* and *SlTCP18*, we used yeast-one-hybrid assays to identify transcription factors binding to the promoter fragments of the ripening related *SlTCP* genes. The promoter elements used were upstream of the transcription start sites and were as follows: 568-bp promoter fragment for *SlTCP12*, 500-bp fragment of *SlTCP15* and 473-bp fragment of *SlTCP18*. In this assay we detected interaction between the ripening regulator RIN and the promoters of *SlTCP12, -15* and -*18* as well as between CNR and the promoter of *TCP18* (Table 2). This data strongly suggests that the regulation of these *TCPs* by RIN and *CNR* is direct.

**Table 2:** Transcription factors binding promoter fragments in a yeast one-hybrid assay

	**promoter fragment**		
**Tomato protein**	***SlTCP12***	***SlTCP15***	***SlTCP18***
SlAP2а	-	-	-
RIN	+	+	+
CNR	-	-	+
SlTCP19	-	+	-
SlTCP11	+	-	-
SlTCP20	-	+	-
SlTCP6	+	-	-
SlTCP9	+	+	+
SlTCP2	-	-	+
SlTCP23	-	+	-
SlTCP18	-	+	-
SlTCP22	-	+	-
SlTCP16	-	+	-
SlTCP1	+	+	-

Representing the binding of: SlAP2a, RIN and CNR, as well as the tomato TCP proteins to the promoter elements of SlTCP12, -15 and -18. ”-” represents no binding, “+” represents binding.

In the cases where regulation of *SlTCP* expression by ripening regulatory transcription factors was detected, but no binding to the target promoter was observed, several other scenarios are possible. The length of the promoter fragments used in the assay is limited and may not have comprised all putative transcription factor binding sites. Alternatively, regulation of expression may be indirect via regulation of expression of other transcription factors that do bind the target promoter.

### Arabidopsis transcription factors binding the promoter fragments of *SlTCP12*, *SlTCP15* and *SlTCP18*

To identify more potential regulators of tomato *SlTCP12*, *SlTCP15* and *SlTCP18*, we performed a yeast one-hybrid assay with transcription factors fused to the GAL4 activation domain. Since there is no large collection of cloned tomato transcription factors, instead we opted for initially testing the available REGIA collection of Arabidopsis transcription factors, consisting of 1397 cloned transcription factor gene open reading frames [41]. All transcription factors interacting with one or more of the promoter fragments are listed in Additional file 4: Table S3. These results demonstrate that the *SlTCP12*, -*15* and -*18* promoter fragments interact with 115, 99 and 86 different Arabidopsis transcription factors, respectively. Nine Arabidopsis transcription factors bind the promoters of all three *SlTCP* genes (Additional file 4: Table S3). One of those is the ethylene-responsive gene *AtDEAR1 (DREB and EAR motif protein 1)*, also named *CEJ1* (*COOPERATIVELY REGULATED BY ETHYLENE AND JASMONATE 1*). In Arabidopsis, *DEAR1* expression is induced by pathogen infection [42].

The common interactions for *SlTCP15* and *SlTCP18* promoter fragments, in addition to the AtDEAR1/CEJ1 protein, are nine. *SlTCP12* and *SlTCP15* share additional 11 common interactions. *SlTCP12* and *SlTCP18* have 32 common interactions, among which one encodes a member of the DREB subfamily A-2 of ERF/AP2 transcription factor family, another ethylene responsive gene. The fact that both *SlTCP12* and *SlTCP18* have more common interactions compared to *SlTCP12* and *SlTCP15* or *SlTCP18* and *SlTCP15*, may be due to their expression overlap during ripening.

This experiment demonstrates that there are a lot of Arabidopsis transcription factors binding the promoter elements of *SlTCP12, -15 and -18*. Whether the same trend can be observed with tomato transcription transcription factors may be further investigated. In the absence of a comprehensive library of cloned tomatos TFs, we speculate that the same would be observed for tomato TFs. Recent studies in Arabidopsis show that many binding sites can be occupied by transcription factors, in a dynamic manner without them necessarily being active regulators of the bound promoter [43]. We performed Gene Onthology (GO-) term enrichment analysis for transcription factors binding to each of the three *SlTCP* promoters versus the tested 1379 *Arabidopsis* transcription factors from the REGIA collection. The results did not show significant enrichment per GO category, suggesting that these TCPs may be involved in or regulated by many different processes.

Some of the *Arabidopsis* proteins binding *SlTCP12* promoter fragment are transcription factors known to be part of the ethylene signal transduction pathway. These include an *ERF/AP2* transcription factor gene encoding for *RELATED TO AP2 3 (RAP2.3)*, other genes from the ERF (ETHYLENE RESPONSE TRANSCRIPTION FACTOR) family, and *ETHYLENE-INSENSITIVE3-LIKE 2 (EIL2*)*.* Several AP2/ERF proteins also bind *SlTCP12*, *SlTCP15* and *SlTCP18* independently (Additional file 4: Table S3). The regulation of *SlTCP* genes by ethylene responsive genes may indicate an indirect mechanism of ethylene-dependant expression of these *SlTCPs* during ripening.

### Tomato transcription factors binding to the *SlTCP12*, -15 and -18 promoter elements

Using the observed interactions with Arabidopsis transcription factors as a lead to candidate regulators from tomato, we selected some of the strongest interacting proteins and identified their closest tomato homologs by Blast homology searches. These were subsequently cloned and used in a yeast one-hybrid assay as described above. The tomato genes used are listed in Table 3 next to their Arabidopsis homologs, together with their interactions with the tomato TCP promoters.

**Table 3:** Binding of Arabidopsis and tomato transcription factors to TCP promoter fragments

		**Promoter fragment**					**Promoter fragment**		
**Arabidopsis protein**	**AGI**	**SlTCP12**	**SlTCP15**	**SlTCP18**	**Corresponding tomato homolog**	**Tomato protein name**	**SlTCP12**	**SlTCP15**	**SlTCP18**
DEAR1/CEJ1	AT3G50260	+	+	+	Solyc04g078640.1		+	-	+
WOX13	AT4G35550	-	+	+	Solyc02g082670.2		-	-	-
KNAT5	AT4G32040	+	+	-	Solyc07g007120.2	LeT12	+	-	-
KNAT4	AT5G11060	+	-	+	Solyc07g007120.2	LeT12	+	-	-
SCL18/LAS	AT1G55580	+	-	+	Solyc07g066250.1	LS	-	-	-
ettin/ARF3	AT2G33860	+	-	-	Solyc02g077560.2	ARF3	-	-	-
AIF3	AT3G17100	+	-	-	Solyc01g058670.2		-	-	-
RVE1	AT5G17300	-	+	-	Solyc02g036370.2		+	-	-
RAV1/EDF4	AT1G13260	-	-	+	Solyc05g009790.1		+	-	-
NAC13	AT1G32870	-	-	+	Solyc12g056790.1		-	-	-
SVP	AT2G22540	-	-	+	Solyc11g010570.1	JOINTLESS	+	-	+
bHLH115	AT1G51070	-	-	+	Solyc07g064040.2		-	-	+
AGL18	AT3G57390	-	-	+	Solyc01g087980.2		-	-	-

Yeast one-hybrid results, representing the binding of the selected Arabidopsis transcription factor proteins and the corresponding tomato putative orthologs to the tomato TCP12, -15, and -18 promoter elements independently. ”-” represents no binding, “+” represents binding.

The Arabidopsis DEAR1, also named CEJ1 binds the promoter fragments of *SlTCP12*, -*15* and -*18* (Additional file 4: Table S3) and its identified tomato homolog Solyc04g078640.1 interacts with the *SlTCP12* and *SlTCP18* promoters (Table 3). LeT12 is the tomato homolog of the Arabidopsis KNAT4 and KNAT5, which bind to the *SlTCP12* and -*15* and *SlTCP12* and -*18* promoter fragments, respectively. The tomato homolog LeT12 however binds only to the *SlTCP12* promoter (Table 3). *LeT12*[44] is a class II *KNOX* gene (*Solyc07g007120.2),* expressed in all tissues, but has high expression in green fruits and leaves [45].

Arabidopsis SVP (SHORT VEGETATIVE PHASE) binds the *SlTCP18* promoter fragment. The tomato homolog of SVP, JOINTLESS (J) binds to both *SlTCP12* and *SlTCP18* promoter fragments (Table 3). In Arabidopsis, *SVP* controls flowering time and maintains the meristematic activity during the early floral meristem stages [46], while in tomato *J* regulates pedicel abscission zone formation and maintenance of the inflorescence meristem [47].

### TCP transcription factors bind the promoters of *SlTCP12*, *SlTCP15* and *SlTCP18*

The protein-DNA interaction assay performed with the Arabidopsis TF collection and the tomato promoter fragments show that there are Arabidopsis TCPs binding the promoter fragments of tomato *TCP12*, -*15* and -*18* (Additional file 4: Table S3). AtTCP6 and -13 bind the *SlTCP18* promoter, while AtTCP1, -3, -4, -8, and -19 bind the *SlTCP15* promoter. The *SlTCP12* promoter did not show any binding activity with *Arabidopsis* TCP transcription factors.

In the yeast one-hybrid assay we also screened for promoter-binding activity of the tomato TCP proteins. This experiment revealed that the binding of *SlTCP12*, -*15* and -*18* by the *Arabidopsis* TCP transcription factors are seen also with most of their tomato SlTCP homologs. SlTCP1, -6, -9, which are class II TCP TFs, bind the class I *SlTCP12* promoter (Table 2). Expression of these proteins peaks in flowers and early fruit development, in contrast to *SlTCP12*, which peaks from mature green fruit onwards and in roots. These complementary expression patterns suggest that binding of the class II TCPs inhibits *SlTCP12* expression. Interestingly, SlTCP11, a class I TCP regulated in all tissues and closest homolog of *SlTCP12*, also binds to the *SlTCP12* promoter (Table 2)*.*

SlTCPs binding the *SlTCP15* promoter are SlTCP1, -9, and -22, which are class II TCPs, strongly expressed in flowers at anthesis and 2 DPA, and in leaves (Table 2). Class I SlTCPs also bind the *SlTCP15* promoter. Among them is *SlTCP18*, whose expression increases during ripening (Table 2). *SlTCP15* shows a different trend of regulation, being regulated by more representatives of its own class. Generally, the proteins from class I and II TCPs binding *SlTCP15* are expressed in flowers, early fruit development and leaves, which fits well with the expression of *SlTCP15*. Therefore, we can hypothesize that this binding leads to activating the expression of *SlTCP15*. The regulation of *SlTCP15* by SlTCP18 may be linked to ripening, because both genes have higher expression during breaker and turning fruit stages.

*SlTCP18* promoter is bound only by class I SlTCPs, which expression patterns are complementary to that of *SlTCP18* (Table 2)*.* This suggests that these class I SlTCPs may regulate *SlTCP18* in a negative way.

**Figure 6:**
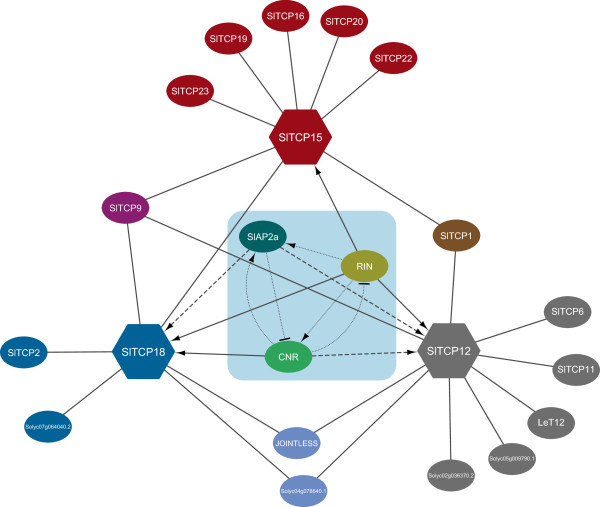
**Network representation of the yeast one-hybrid detected interactions.** The network was generated by Cytoscape v3.0.2, and shows the identified interactions between the tested DNA baits (nodes represented by hexagons) and the detected TF interactors (nodes represented by ovals), with interactions represented by solid edges. The solid arrows represent direct interactions and dashed arrows represent indirect interactions. The direction of these interactions was identified by expressions analysis of *SlTCP12*, *SlTCP15* and *SlTCP18* in the tomato ripening mutants *rin*, *Cnr* and RNAi plants suppressing *SlAP2a*. Dotted arrows represent interactions, reported in literature.

The protein-DNA interaction between TCP transcription factors and *SlTCP12,-15 and -18* promoter elements suggest that TCPs regulate the expression of other members of their own family. This regulatory network is visualized in Figure 6. It also suggests that SlTCPs from class II regulate *SlTCPs* from class I, and SlTCPs from class I regulate *SlTCPs* from the same class. This cross-regulation among *SlTCP* genes suggests that class II SlTCPs may act as repressors of class I, but on the other hand, class I may activate genes from their own class, as this may be the case with *SlTCP15*. Thus, the tomato *TCP* genes are likely part of an interrelated regulatory network, as has already been described for *TCP* genes in Arabidopsis [7]. Since these regulatory interactions were inferred from yeast one-hybrid assay results, which are prone to producing false positives, further *in vivo* and/or *in planta* experiments are needed to confirm these interactions.

## Conclusions

In conclusion, we have identified the 30 members of the tomato *TCP* transcription factor gene family. Tomato genes closely related to Arabidopsis *TCP* genes, have similar expression patterns, which suggests conserved functions. Additionally, the tomato TCP proteins form homodimers and heterodimers particularly with SlTCPs from the same class (Figure 4). This trend was reported before also in other species, like Arabidopsis [35] and rice [25]. SlTCP19 is an exception, because it is a class I SlTCP, dimerizing only with class II SlTCPs (SlTCP1 and SlTCP2). Interestingly, the tomato *TCP12, TCP15 and TCP18* genes show differential expression patterns during fleshy fruit development and ripening. Expression studies show that *SlTCP12*, -*15* and -*18* are positively regulated by the ripening regulators RIN, CNR and SlAP2a, which are among the promoter binding proteins of these TCP genes (Figure 6). These data shows that the *SlTCP12*, -*15* and -*18* genes are directly or indirectly controlled by these ripening regulators and might play a role in tomato fruit ripening. Furthermore, we show that SlTCP proteins can bind to the promoter sequences of other *SlTCP* genes (Figure 6), suggesting that they could coordinately or competitively regulate their expression. Our promoter and protein-protein interaction studies suggest that *SlTCP12*, -*15* and -*18,* may be involved in a variety of other functions, in addition to fruit ripening. Further research analyzing the phenotypes of knockout or knock down of SlTCP12, -15 or -18 will reveal more information about the function of these genes.

## Methods

### Plant Material

*Solanum lycopersicum* 'Moneymaker' was used as source for plant material. Seedlings were grown on agar culture for 21 days. For the collection of roots, seedlings were grown along a Whattman filter paper and fed with 0.5 MS medium for 28 days. For the other samples, plants were grown in the greenhouse and the following tissues or organs were harvested: fully expanded leaves, flowers at anthesis, flowers two days post anthesis, developing fruits with 5 mm diameter, developing fruits with 18 mm diameter, mature green fruits, fruits at breaker stage, fruits at turning stage and fruit at red ripe stage. Plant materials were frozen in liquid nitrogen and ground with IKA A11basic (Staufen, Germany). The material was stored at -80°C until further use.

### TCP sequence identification and cloning

In order to identify tomato genes putatively encoding TCP transcription factors, the Solanaceae Genomics Network (SGN)-Unigene database (for ESTs) as well as the BAC sequences deposited by the International Tomato Sequencing Project were searched using the TBLASTN algorithm with Arabidopsis TCP proteins or TCP domains as query sequence. For thus identified unigenes, EST clones, when available, were obtained from the Boyce Thompson Institute for Plant Research at Cornell University (Ithaca, New York) and resequenced to confirm identity and to establish the presence or absence of 5’ and 3’ ends of an open reading frame. Where either one or both open reading frame ends were not present or where no EST was available, mRNA sequence was extended by 5’ and/or 3’ RACE, as was done for three partial cDNA sequences deposited earlier in GenBank (AAO45726, AAO45727, AAO45728 [31]). For three putative *TCP* gene sequences found in BAC sequences only, the genomic DNA sequence was taken as template for the design of RACE primers. For this purpose, RNA was isolated of tomato fruit pericarp of 7 developmental stages (7 days after pollination to red ripe). mRNA was isolated using the RNAeasy Plant MiniKit (50) (QIAGEN). All RNA’s were mixed together in equal amounts. 5’and 3’ RACE-ready cDNA libraries were made from RNA using the Clontech SMART RACE cDNA amplification kit (Westburg B.V., Leusden, the Netherlands). Full-length open reading frames were amplified by PCR using ESTs (when containing the entire orf), 5’ and 3’ RACE clones, or genomic DNA. Genomic DNA was isolated from leaves using an earlier described method [48]. All sequencing and open reading frame amplification templates, as well as primers used for RACE PCR and open reading frame PCR are listed in Additional file 2: Table S4 and S5. Open reading frames were amplified using primers with a 5’ CACC extension, and purified using the QIAquick PCR Purification Kit or purified from gel using the QIAquick gel extraction kit (Qiagen) and ligated between GATEWAY *att*L1 and *att*L2 sites in pENTR/D-TOPO using an Invitrogen pENTR™ Directional TOPO® Cloning Kits (http://www.lifetechnologies.com), producing GATEWAY Entry vectors. Ligated products were transformed into *E. coli* DH5α competent cells by electroporation. All entry clones were checked by sequencing analysis (BigDye sequencing kit, Applied Biosystems). (DYEnamic ET Terminator (Dett) Cycle Sequencing Kit from Amersham Biosciences, GE Healthcare). The messenger RNA sequences of the first identified 24 SlTCPs have been deposited in GenBank under accession numbers listed in Table 1.

### Phylogenetic analyses

Tomato TCP protein sequences were compared with all 24 *A. thaliana* TCP proteins. Multiple sequence alignment was performed with Muscle [49] as implemented in MEGA v5.10 [50]. Phylogenetic reconstruction was obtained by the NJ (neighbor-joining) method [51] using the Jones-Taylor-Thornton (JTT) substitution model with Gamma-distributed rates (5 categories) among sites together with bootstrap analysis using 500 replicates.

### Gene expression analysis

Extraction of total RNA was performed with the use of TriPure reagent (Roche Diagnostics, Indianapolis, USA) according to the manufacturer’s protocol. DNAase treatment was performed with DNAase I (Invitrogen, Breda, the Netherlands) according to the protocol. RNeasy columns (Qiagen, Venlo, the Netherlands) were used to purify the RNA. Quantitative and qualitative concentration measurements were performed using Nanodrop ND-1000 Spectrophotometer (NanoDrop Technologies, Delaware, USA).

For determination of transcript concentrations, quantitative real time RT-PCR was performed in two biological replicates. Briefly, 1 μg of total RNA was used for cDNA synthesis using the TaqMan Reverse Transcription Reagents kit (Roche Molecular Systems, Branchburg, USA). Primers were designed using Beacon Designer (Biosoft International, Palo Alto, USA) and purchased from Biolegio (Nijmegen, The Netherlands). Real-Time PCR was performed in a MyIQ Single-Color Real-Time PCR Detection System (Bio-Rad, Veenendaal, The Netherlands) using the following program: 3 min denaturation at 94°C, 40 cycles of 15 sec at 94°C and 30 sec at 60°C, followed by a melting curve gradient to analyze the specificity of the primer pairs for a particular gene. No Template-Controls served as blanks and β-Actin was used as reference gene based on least variation observed in the 11 tomato tissues. For the ripening mutants, *Cnr*, *rin*, *nor*, and the knock down *SlAP2a*, RNA from Breaker + 7 was isolated using an InviTrap® Spin Plant RNA Mini Kit (http://www.invitek.de). The RNA quality was checked on gel and cDNA was synthesised using TaqMan® Reverse Transcription Reagents (Invitrogen™) with 1 μg of RNA. The qRT-PCR was performed on BIORAD iQ5 using SYBR-green fluorescence dye. The program used was as described before for the TCPs qRT-PCR.

Sequences of the primer pairs used are listed in Additional file 2: Table S6. C_t_-values of the 11 samples were measured in duplo and averaged, followed by calculation of the relative gene expression using the 2 ^-δCt^ method for the expression of *SlTCP* transcripts in the different tomato organs and the 2 ^-δδCt^ method [52] for the expression of *SlTCPs* in the ripening mutants. Analysis of the reaction efficiency was performed with the LinRegPCR program [53].

### Yeast two-hybrid assays

All TCP ORFs were recombined from the entry clone into the pBDGAL4 bait vector (pDEST™32, Invitrogen) and pADGAL4 prey vector (pDEST™22, Invitrogen). The bait vectors were transformed into yeast strain PJ69-4α (*MAT*α) and all prey vectors into strain PJ69-4a (*MAT*a [54]) and selected on SD plates lacking Leu and Trp, respectively. Subsequently, overnight cultures were grown (30°C, 300 rpm) from single colonies of each transformant in selective SD medium and systematically mated with each other by spotting 5 μL droplets of the liquid cultures on top of each other on SD complete plates (Nunc Omnitray; VWR International, Amsterdam, the Netherlands) containing all the essential amino acids. In addition, some negative control combinations were spotted, for which water was used instead of either a bait or prey culture. Subsequently, the plates were incubated at 30°C for 16 h, and afterwards the yeast was transferred to SD plates lacking both Leu and Trp to select for diploid yeast containing both plasmids. After 2 d of growth at 30°C, the yeast was transferred to four different selection plates containing SD medium lacking Leu, Trp, and Ade (-LTA) and SD lacking Leu, Trp, and His (-LTH), supplemented with 5, 10, or 15 mM 3-amino-1,2,4-triazole (3-AT), respectively. Bait clones were tested for autoactivation in the absence of an interacting prey protein by plating on SD lacking Leu and His supplemented with increasing concentrations of 3-AT. For subsequent interaction experiments only growth on selective media (if any), where no growth of autoactivating clones occurred, was scored. These plates were incubated at 20°C and scored for growth of yeast and hence protein–protein interaction events after 5 d. The screening was performed in triplicate. In case of autoactivation for one of the two proteins, just four data points were obtained for the specific combination. The mating efficiency appeared to be 100%, and where water was used for mating, either instead of a bait culture or instead of a prey culture, no growth was obtained on medium selecting for the presence of the two plasmids or on the media selecting for interactions. This shows that no cross-contamination occurred as a result of the procedure that followed. A combination was scored as a true interaction when it resulted in growth for at least one of the two selection markers (Adenine or Histidine) in at least three out of four experiments. Combinations that grew only on one selective medium were marked as such in the presentation of the results.

### Yeast one-hybrid assay

DNA-protein interactions between Arabidopsis transcription factors and the single tomato TCP12, TCP15 and TCP18 proteins were identified and characterized using yeast-one hybrid assay, which was based on Clontech’s Matchmaker Gold Yeast One-Hybrid (Y1H) System (http://www.clontech.com). This system uses the antibiotic Aureobasidin A resistance as a reporter. PJ69-4A yeast strain was used for the transcription factor baits and PJ69-α for the TCP12, -15 and -18 promoter reporter constructs. The single promoter fragments were cloned into the pAbAi reporter vector, which was made Gateway compatible. Primers used for the promoter elements cloning can be seen in Additional file 2: Table S7. The reporter construct of TCP12 consists of 568-bp (region SL1.03sc00008:1785048..1785615), TCP15 – of 500-bp (region SL2.31sc04133:30626689..30627956) and TCP18 – of 473-bp (region SL1.03sc01076:4199969..4200441). For each of the promoter fragments, an autoactivation test was performed with Aureobasidin A concentrations ranging from 0 to 500 ng/ml. The Y1H screenings were done at concentration of 75 ng/ml Aureobasidin A for each reporter, which was the lowest background activation detected. The yeast clones were grown and selected for the presence of the plasmid for 2 to 3 d on selective medium at 30°C. The transcription factors bait and the TCPs reporter clones were mated on SD complete medium overnight and then transferred onto bait/reporter selection medium for 3 days. The media-selected mated yeasts were transferred to 100 μL of sterile MilliQ water and 5-μL droplets were spotted onto aureobasidin-containing plates. Plates were incubated at 20°C and scored after 5 to 7 days.

The transcription factors (TFs) library (The REGIA TF ORF Library) used for the yeast one-hybrid screen contains a set of *Arabidopsis thaliana* transcription factor open reading frames (ORFs) [41]. After identification of the protein – DNA binding, the Arabidopsis TFs, which were found to strongly bind the promoter of the studied tomato TCP proteins, were characterized as candidate genes. The selection of the tomato candidate TFs was based on their closest Arabidopsis homolog, characterized by protein - nucleotide blast search in the database of SOL Genomics Network. Some of the tomato candidate genes, which had expression during fruit development and ripening (based on EST and Unigene expression data) and the tomato homologs of the strongest bound Arabidopsis TFs were selected and cloned (Additional file 4: table S3).

### Cloning of the tomato candidate TFs used in the yeast one-hybrid screen

The ORFs of the single tomato candidate TFs were amplified and independently cloned in pCR™8/GW/TOPO vector (Invitrogen). All entry clones were controlled by sequencing analysis, then recombined into the pADGAL4 vector (pDEST™22, Invitrogen) and transformed into PJ69-4A yeast strain [55]. In summary, 40 tomato open reading frames were cloned (Additional file 2: Tables S5 and S8). The yeast one-hybrid experiment was performed in triplicate with the tomato TFs.

### Cytoscape - network data integration, analysis and visualization

Cytoscape v3.0.2 [56] was used for generating network of the yeast-one hybrid interactions and for integrating the expression data of *SlTCP12*, -*15* and -*18* in the ripening mutants *Cnr*, *rin* and the *SlAP2a* knockdown plants. Default settings were used with nested network style.

BiNGO 2.8 plugin for Cytoscape [57] was used for GO term enrichment analysis. In the search for overrepresentation we used the standard settings: Benjamini-Hochberg FDR, significance level of 0.05.

### Availability of supporting data

The data sets supporting the results of this article are included within the article and its additional files (additional figures and tables).

## Competing interests

The authors declare that they have no competing interests.

## Authors’ contributions

Authors, who have made substantial contributions to conception, design of experiments, acquisition of data, analysis and interpretation of data: VP, RD, AB, RK. Authors who have contributed to performing experiments: VP, ML, MB, JB. Authors who have been involved in drafting the manuscript: VP and RM. Authors who have revised it critically: VP, RM, RK, GA. Authors who have given final approval of the version to be published: all. Authors who agree to be accountable for all aspects of the work in ensuring that questions related to the accuracy or integrity of any part of the work are appropriately investigated and resolved: all.

## Supplementary Material

Additional file 1: Figure S1 Chromosomal location of the tomato TCP genes. “1a-d”, “19a:, and “28a” depict the extra copies of the respective genes in the tomato reference genome. Click here for file

Additional file 2: Table S1 Additional (partial) copies of *SlTCP1*, *-19*, and *-28* found in the tomato reference genome. **Table S4.** Tomato TCP genes and TCP-like sequences, corresponding SGN-Unigenes, (re) sequenced EST clones, and RACE experiments performed to obtain full-length cDNAs. **Table S5.** Sequences of primers used for amplification of open reading frames. **Table S6.** Sequences of primers used for quantitative RT-PCR. **Table S7.** Primers used for amplification of TCP12, TCP15 and TCP18 promoters. **Table S8.** Primers used for the amplification of the ORF of tomato candidate genes. Click here for file

Additional file 3: Table S2 Scoring tables for the yeast two-hybrid experiments. Every experiment was performed in triplicate and interactions were scored as positive if growth occurred on both selection media in at least two of the three experiments. Click here for file

Additional file 4: Table S3 Yeast one-hybrid results, representing the binding of the Arabidopsis transcription factor proteins and the cloned corresponding tomato homologous proteins to the tomato TCP12, -15, and -18 promoter elements independently. ”-” represents no binding, “+” represents binding. Click here for file
