# Supplementary figures and images for: Identification, cloning and characterization of the tomato TCP transcription factor family
Source: BMC Plant Biol. 2014 Jun 6;14:157. doi: 10.1186/1471-2229-14-157 (PMC4070083; doi:10.1186/1471-2229-14-157)

0

1

2

3

4

5

6

7

8

9

10

11

12

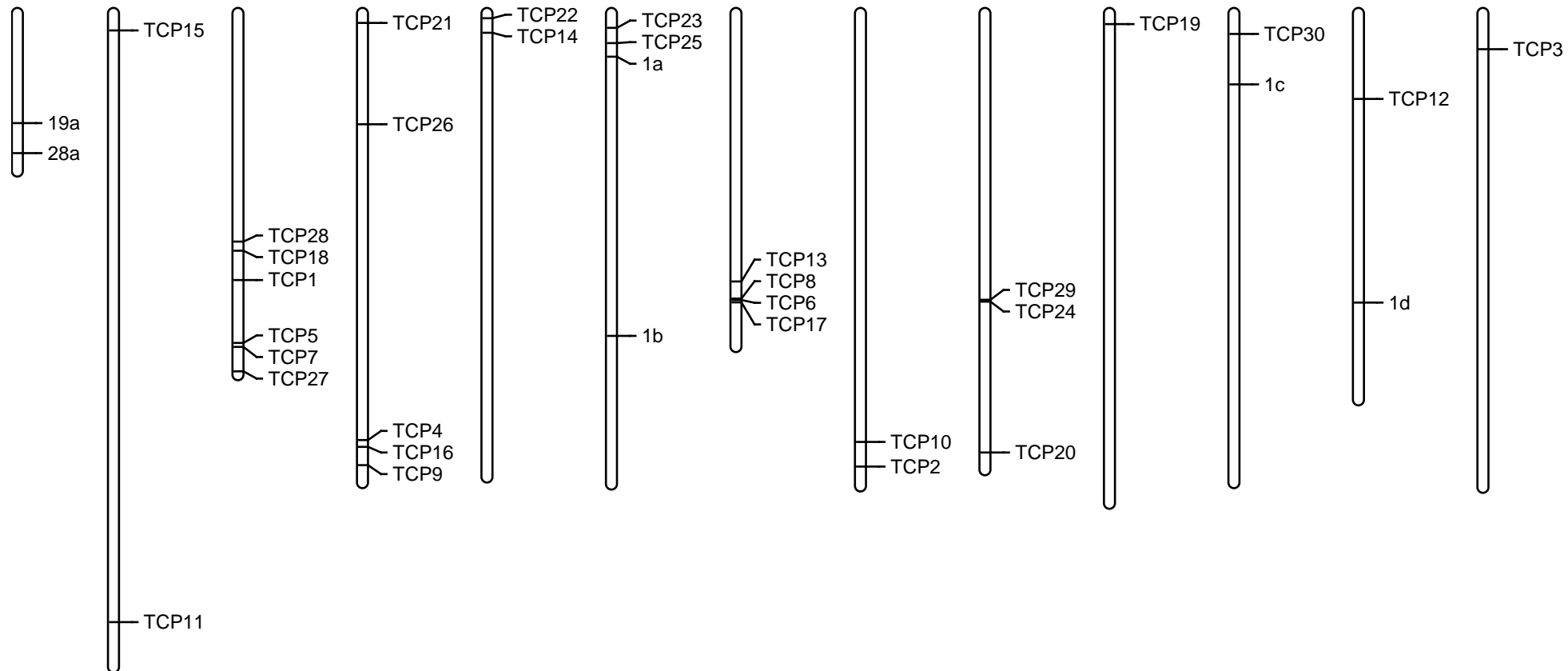

Supplement: Additional file 1: Figure S1 — Chromosomal location of the tomato TCP genes. “1a-d”, “19a:, and “28a” depict the extra copies of the respective genes in the tomato reference genome. [file 1471-2229-14-157-S1.pdf]
