# Supplementary material for: Identification, cloning and characterization of the tomato TCP transcription factor family
Source: BMC Plant Biol. 2014 Jun 6;14:157. doi: 10.1186/1471-2229-14-157 (PMC4070083; doi:10.1186/1471-2229-14-157)
Supplement: Additional file 2: Table S1 — Additional (partial) copies of SlTCP1, -19, and -28 found in the tomato reference genome. Table S4. Tomato TCP genes and TCP-like sequences, corresponding SGN-Unigenes, (re) sequenced EST clones, and RACE experiments performed to obtain full-length cDNAs. Table S5. Sequences of primers used for amplification of open reading frames. Table S6. Sequences of primers used for quantitative RT-PCR. Table S7. Primers used for amplification of TCP12, TCP15 and TCP18 promoters. Table S8. Primers used for the amplification of the ORF of tomato candidate genes. [file 1471-2229-14-157-S2.pdf]

Additional Table 1. Additional (partial) copies of *SlTCP1*, *-19*, and *-28* found in the tomato reference genome

Name[a]	Gene (ITAG2.4)	SGN Unigene
SlTCP1-like a	Solyc05g012840.1	None
SlTCP1-like b	Solyc05g032780.1	None
SlTCP1-like c	Solyc10g018710.1	None
SlTCP1-like d	Solyc11g045640.1	None
SlTCP19-like a	Solyc00g084870.2	None
SlTCP28-like a	Solyc00g217560.1	None

Additional Table 2. Scoring tables for the yeast two-hybrid experiments. Every experiment was performed in triplicate and interactions were scored as positive if growth occurred on both selection media in at least two of the three experiments.

File can be found under name and format: "Additional Table 2.xlsx"

Additional table 3. Yeast one-hybrid results, representing the binding of the Arabidopsis transcription factor proteins and the cloned corresponding tomato homologous proteins to the tomato TCP12, -15, and -18 promoter elements independently. "-" represents no binding, "+" represents binding

File can be found under name and format: "Additional Table 3.xlsx"

Additional table 4. Tomato TCP genes, corresponding SGN-Unigenes, (re)sequenced EST clones, and RACE experiments performed to obtain full-length cDNAs

Name	Gene (ITAG2.4)	SGN Unigene	Sequenced EST clones[a]	RACE[b]
SlTCP1	Solyc02g077250.2	none		5', 3'
SlTCP2	Solyc07g062680.1	SGN-U594432, SGN-U566833	cTOC19N10, cLEB3N9	-
SlTCP3	Solyc12g014140.1	SGN-U594431, SGN-U566832	TUS60P6, cLET3M23, cLEL35O17, cTOF34G9	-
SlTCP4	Solyc03g115010.1	SGN-U582556, SGN-U603841		5', 3'
SlTCP5	Solyc02g089020.1	SGN-U585799, SGN-U585800	cLET3H17, cTOC20G10, cTOC22M17	5'
SlTCP6	Solyc06g069460.1	SGN-U601349	cTOA29H21	-
SlTCP7	Solyc02g089830.1	None		5', 3'
SlTCP8	Solyc06g069240.1	None		5', 3'
SlTCP9	Solyc03g119770.2	SGN-U584141	cTOS6K18	5', 3'
SlTCP10	Solyc07g053410.2	SGN-U597981	cTOF6D9	5'
SlTCP11	Solyc01g103780.2	SGN-U577117	TUS37P1, cTOF17M2, cTOC22M8	5'
SlTCP12	Solyc11g020670.1	SGN-U573569	TUS24K10	5'
SlTCP13	Solyc06g065190.1	SGN-U562794	cTOC4H22, cLEI13H13	-
SlTCP14	Solyc04g009180.1	SGN-U579351, SGN-U563595	TUS61K24, TUS69A20	-
SlTCP15	Solyc01g008230.2	SGN-U581159	cTOF6B9, cTOC12D24, cTOC24I7	5'
SlTCP16	Solyc03g116320.2	SGN-U574962, SGN-U571327	cTOF23G15, cTOF31L2	-
SlTCP17	Solyc06g070900.2	SGN-U586610	cLEI9N14	-
SlTCP18	Solyc02g068200.1	SGN-U576961	cTOC20P22	-
SlTCP19	Solyc09g008030.1	SGN-U567380	TUS60E22, cLES12D23	3'
SlTCP20	Solyc08g080150.1	SGN-U566570	cLED17B2, TUS22B5	5', 3'
SlTCP21	Solyc03g006800.1	SGN-U603785	cTOC1O20	5', 3'
SlTCP22	Solyc04g006980.1	SGN-U583072, SGN-U602382	TUS69G5	-
SlTCP23	Solyc05g007420.1	SGN-U603706		5', 3'
SlTCP24	Solyc08g048390.1	SGN-U573225	cTOF3D1	3'
SlTCP25	Solyc05g009900.1	None	from gene model	
SlTCP26	Solyc03g045030.1	None	from gene model	
SlTCP27	Solyc02g094290.1	SGN-U599631	from gene model	
SlTCP28	Solyc02g065800.1	None	from gene model	
SlTCP29	Solyc08g048370.2	None	from gene model	
SlTCP30	Solyc10g008780.1	SGN-U603276	from gene model	

[a]EST clones (re)sequenced for finding clones containing a full open reading frame
[b]RACE experiments performed for de novo sequencing of transcripts and for obtaining templates for the amplification of the open reading frame

Additional table 5. Sequences of primers used for amplification of open reading frames

Name	Forward primer	Reverse primer
SlTCP01	CACCCAGCAGATGCCACAGTCACTCTGC	GGGTCTCAAGTTATTTGCTTAATGGATC
SlTCP02	CACCATGGCAGAAACGTCAAGATTGGGTATA	AACTTTTTGGAGCTAATTCAATGGCG
SlTCP03	CACCGATCATTTTATGTATGAAGATTTAGATACCATC	TTCAATGACGAGAATCAGAGGAAGC
SlTCP04	CACCATGAATTCATCGAGTGGAAATAATTTTG	GGCTTAGAATCAAATTGCGCGACC
SlTCP05	CACCATGATTACAAGAGAAGAGAAGGGGAAC	GATCTTAATTGGAAGGCTCCGTGTCATCAC
SlTCP06	CACCATGAACTCAAGAAAGCAAGAAACTG	ATATGCTTAAGAACCACTGCCTTC
SlTCP07	CACCATGTTCCCTTTTGGAAACAGTAGTAATGGTGG	GCTAGCTTACGCTGTTCCCTTCAGA
SlTCP08	CACCATGTATCCTCCAAGCAACAATAACTGCAGC	CCTTGCCCTAGCCTTCCCATAGC
SlTCP09	CACCATGTACCCTTCGAGCAATTACAGCCC	TTAGCCTTGAATACCTGATTGATGATC
SlTCP10	CACCATGAAGAGCGCAACTGGAGGA	AATGATGAGCATTAGGAGAAGCAGAG
SlTCP11	CACCCCCAAAATTATGGAACTCACTGATTTAC	CTTTCAAGCACTTTCACTCCATCACACTAGAG
SlTCP12	CACCAAAATGGAATTCATGGAACCACATAC	CATCGAGATTTGTAAACTATGATTG
SlTCP13	CACCGCCATGGAATTTAGAGAGTCAAATAG	GTTGGAGTTATGATTGGGAATCTTTGTG
SlTCP14	CACCATGTCGACGTCTGCAGAGGGAAA	TCAACGTCCGTCGTCGTCTCTT
SlTCP15	CACCATGTCGACGTCGGTAGAGCAAAAC	CTTCATTGTCCATCGTCGTC
SlTCP16	CACCGTGAACATGGATGGAGGAGGTGATG	GCTATGAATGATGGCTCGTTGTATCG
SlTCP17	CACCATGGAAGGAGGAGGAGGTGACG	TTAGGAATTATGATGGCTTGTTGAATC
SlTCP18	CACCATGGATCCCAAACAGG	ACCAAATGAAAAATCATTCACC
SlTCP19	CACCATGGGTTCAGAAATAGTTGTTTCCGGT	CTCTACTGTTTCTCATCTTCTTCGGCG
SlTCP20	CACCATGTCCAGTTTTCATGACCCAGATG	CTATGAACGTTCTGAAGATGTAACTTGAG
SlTCP21	CACCAAAATGGCGAGGGTTGAGAACA	TCAGGATTTAGATGAGGAAGTTTG
SlTCP22	CACCCTAAACATGTTTCCTTCAAGCAATA	CGACTTGAAACTTATTCCAATCTATGATGT
SlTCP23	CACCATGGAAACTGGCCATCATCATCATG	TCAAGAATGATGTTGATGATCATCTCC
SlTCP24	CACCAGTATGGAGATGGAGGAGATTCG	GCCATGGAAGTTTAGTTCTTTC

Additional table 6. Sequences of primers used for quantitative RT-PCR

Name	Forward primer	Reverse primer
SlTCP01	AGCAAGAGGTGAATTATGACATCC	CTGAAGTGGTGGTGAAGAAGC
SlTCP02	CTCTGATTCTCGCCATTGAA	TCTCCATCTCCAGGCTCTTT
SlTCP03	CAATTGCTGGAGCACGTAGT	GCACGAATCGAAGGTGAGT
SlTCP04	CGATACTCTTCCTCCACTTC	AGCGATTGACTTGTTCCTC
SlTCP05	CGGAGCCTTCCAATTAAGAT	ATGAAACATCCGATCAACCA
SlTCP06	TCAACAGCAACAACAACAACA	CCCTGAACCAGTTTCTTGCT
SlTCP07	ATCGGAGGGTGAGGTTGTC	AACCAAGCATGTCTTGCAAAT
SlTCP08	TGGTGCAATTTGTGCATCTA	ATCTTGAGCGGTTTCCTTGT
SlTCP09	GAATTCGGGTTCATATTGCAT	AAAGATGTACCCTTCGAGCAA
SlTCP10	CGCCACCAACACAATCAC	ATAGCAAGAGAAGTAGGTATAAGC
SlTCP11	GCCACTAGATCGGACACCTT	GCTGCACAGGTTGTGTTTGT
SlTCP12	GACCACCAATTGTGGCAGTA	AAACTCCAACCCACCAATTC
SlTCP13	GGCAAGACCCAGATGAGAAT	CCTTGAACTCCGGTTCTTTC
SlTCP14	GGAATTACTTACCAATGACTC	ACAACAATAACGATAACAATAAC
SlTCP15	GGCGTAGGAGGAGGAGGAGAG	TCGTCGGCAGAGGCATAACC
SlTCP16	ATCCGATGGCGAAACAATAG	GTTCCTGTAGCGGCGATTAC
SlTCP17	AAGACCGCCACACTAAGGTT	ATCGGATTTGTGACCGAGTT
SlTCP18	AATCCTCATCTGCTCCATCA	GTAGCTGCAGGCAGATCAAA
SlTCP19	CCTACCGGAAATCCAGAAAC	CGCTGCTACAGGAACAGGTA
SlTCP20	AACCGTTTGATTCCGATCAT	CTACCGGCATAGGCATTGAT
SlTCP21	ACATTGAACAAGAAGATGACGAG	CCTTTAACAACAGCACCCTATC
SlTCP22	AACCTAAGTAAGCCATCATCAC	GCTCAAGGGAGACTCATTATG
SlTCP23	AACCAACAACAACAAAGGATAGAC	ACCAGTAGCAGCAATAACAGC
SlTCP24	TAGACGTGTTCGCCTTTCTG	AGAGGTGGAAGCTCAGCAAT
SlTCP25	GGGATCATGGAAAGGTACTTAGG	CAAGAATCCCCATATTGAAGC
SlTCP26	ACACTCCGAATTAGCCCTTG	CTGAAATTTTCGGGCGATG
SlTCP27	GCTATTACAGAAAGCGGAACC	GTAAAGCGATGCCTGAATTG
SlTCP28	CATCATCGTCATCATGGACA	GCTCGTGACACACGAACAAT
SlTCP29	ACCGAAATATGCACCCTAGC	CTCGGTGTGTTTTGGTCATC
SlTCP30	GCACAGCAAGGTTTCAACAG	CTGAATTGCCGTGTTAGGAG

Additional table 7. Primers used for amplification of TCP12, TCP15 and TCP18 promoters

Name	Forward primer	Reverse primer
SlTCP12	CCTACACCACCATAATCGCTTC	AGTGAATTTAGAAGGAGCTGTCC
SlTCP18	TCAGGACTCTATTAAAATCAAAATGC	TCAGTAGCAATAGTGTGGCTAAACC
SlTCP15	CACCATCGAAATGACAAATACTC	GATTTTTATGGAAAAATAAACACCAA

Additional table 8. Primers used for the amplification of the ORF of tomato candidate genes

SGN-Unigene	Name	Forward primer	Reverse primer
SGN-U571928	*LeT12*	ATGGAGTTTCAGGACCACTT	TTACTGCTTGATTTCACCTG
SGN-U580795	*ARF 3*	ATGATGTGTGGACTTATTGATCTGA	CTACAGAGCAATATCAAGAAGCACA
SGN-U566000		ATGGGTAGAGGAAAAATTGACATAA	TCACTTTGAGCCAACTTGATTCTC
SGN-U578103	*JOINTLESS*	ATGAAACAAATTCTTGAGAGGC	TCAGCCTGAGTAAGGTAGCC
SGN-U575250		ATGGTTTCACCGGAGAGTA	TTAAGCAACTGGAGGACGG
SGN-U585288		ATGAAGATGTTTGAGTTATCTGATTC	TTAGCAGCAGCAGAGCTCTTG
SGN-U563109		ATGGATTGGGAAAAACAGCA	TTAGCCGGTCATGTTGTAGTC
SGN-U573409		ATGGAAGGAAGTATTAGTAGCATAGATCA	TACAAAGCATCAATAATAACCCTTTG
SGN-U562646		ATGGAAGGAGAGAAGAGAAAACA	TCAGTTATCTTCGTCGCAACTT
SGN-U602159	*LATERAL SUPRESSOR*	ATGTTAGGATCCTTTGGTTCTTCA	TCAACGCCAAGACGAGATG
SGN-U579452		ATGTCTTCAACGGTGATTTC	TCAAATAGGAGGAGGTCGA
SGN-U563553		ATGACTATAGGGGATCAAAGCG	CTACAAACATAATCGGGTTCGTT
SGN-U583072	*SlTCP22*	ATGTTTCCTTCAAGCAATAACCA	TTATTCCAATCTATGATGTTTGGAGGA
SGN-U591168	*AP2a*	CACCATGTGGAATTTAAATGATTC	AGGTCTCATAAAATAATGATGGA
SGN-U578471	*RIN*	CACCATGGGTAGAGGGAAAGTAGAATTGAAG	TCAATGCCATGATGTTTGAAAGG
SGN-U576709	*CNR*	CACCATGGAAACTAACAAATGGGAAG	GCCCAAATTTTCTCCATGAGAG
